# Supplementary material for: Premature Cdk1/Cdc5/Mus81 pathway activation induces aberrant replication and deleterious crossover
Source: EMBO J. 2013 Mar 26;32(8):1155–67. doi: 10.1038/emboj.2013.67 (PMC3630363; doi:10.1038/emboj.2013.67)
Supplement: Supplemental Information [file emboj201367s1.pdf]

The supplementary information contains 6 figures, 1 table and Extended Experimental Procedures.

Figure S1 Szakal and Branzei, 2013

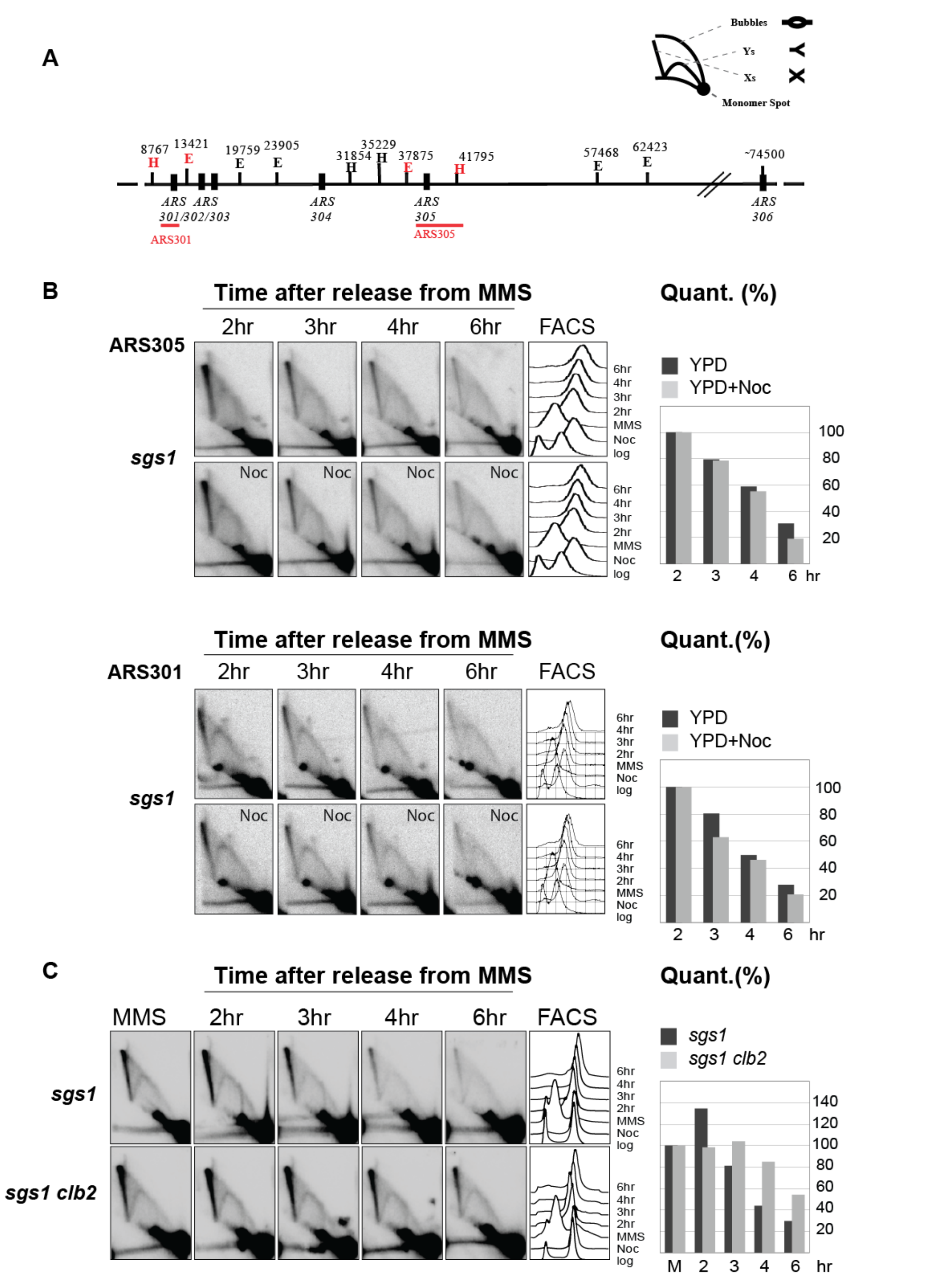

Figure S1. DDT intermediate resolution in G2/M. (**A**) Schematic representation of replication intermediates visualized by 2D gel electrophoresis and of the genomic region containing the *ARS301* and *ARS305* origin on chromosome III. E and H stand for *Eco*RV and *Hind*III, respectively. (**B**) DDT intermediate resolution in G2/M occurs largely before anaphase. G2 synchronized *sgs1* (HY0501) cells were released in media containing MMS and then released in YPD media with or without nocodazole (Noc). Samples were taken for 2D gel and FACS analysis after the release from MMS at the indicated time-points. Replication intermediates were visualized using *ARS305* specific probe. Then filters were stripped and re-hybridized with an *ARS301* specific probe. The value obtained for X-shaped intermediates in 2h samples was considered as 100% and the other values were normalized to it. (**C**) Mitotic cyclins and Cdk1 activity are required for the late resolution. G2 synchronized *sgs1* (HY0764) and *sgs1 clb2* (HY2039) cells treated with MMS and released in YPD media for recovery. Samples for FACS and 2D gel analysis were taken at the indicated time points. The relative value for the X-molecule signal accumulated after MMS treatment was considered as 100% during quantification.

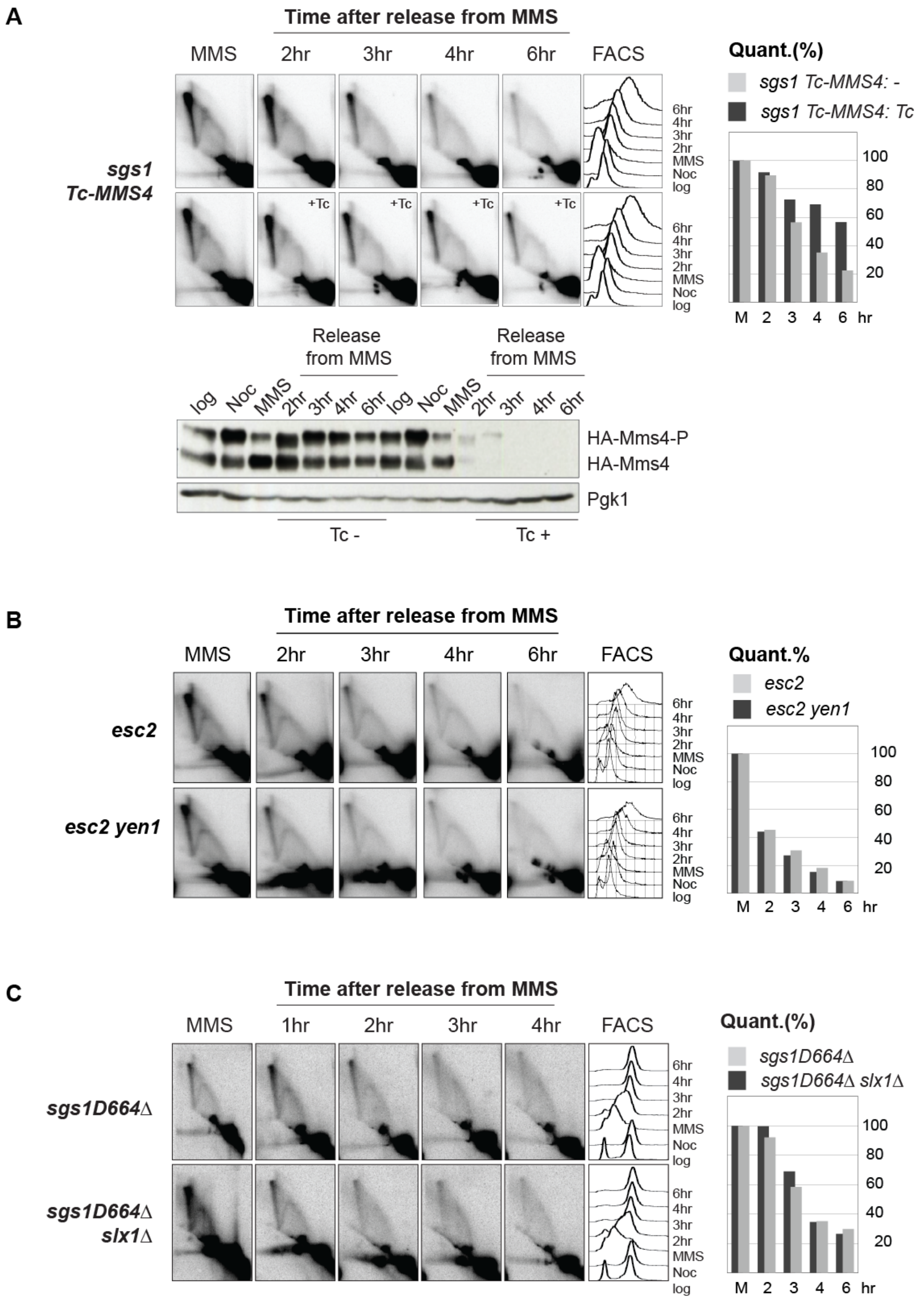

Figure S2. Enzymatic activities required for the late resolution. (**A**) DDT intermediate resolution in G2/M depends on the Mus81-Mms4 endonuclease. G2-synchronized *sgs1 Tc-MMS4* (HY1947) cells were released in media containing MMS for 90 min, then allowed to recover in YPD media containing or not tetracycline (Tc). Mms4 (tagged with 3HA) levels were analyzed by Western blot. Pgk1 was used as a

loading control. For both panels, the relative values obtained for the X-molecules accumulating after the MMS treatment were considered as 100%. **(B-C)** Yen1 and Slx1 nucleases are not required for the late resolution. *esc2Δ* (FY1081), *esc2Δ yen1Δ* (HY2055), and *sgs1-D664*Δ (FY1083), *sgs1-D664*Δ *slx1Δ (*HY3244) cells were synchronized in G2, treated with MMS 0.033% for 90 min, then allowed to recover in YPD media. Samples were taken at the indicated time points for FACS and 2D gel analysis. For each strain, the relative value for the X-molecule signal accumulated during MMS treatment was considered as 100%.

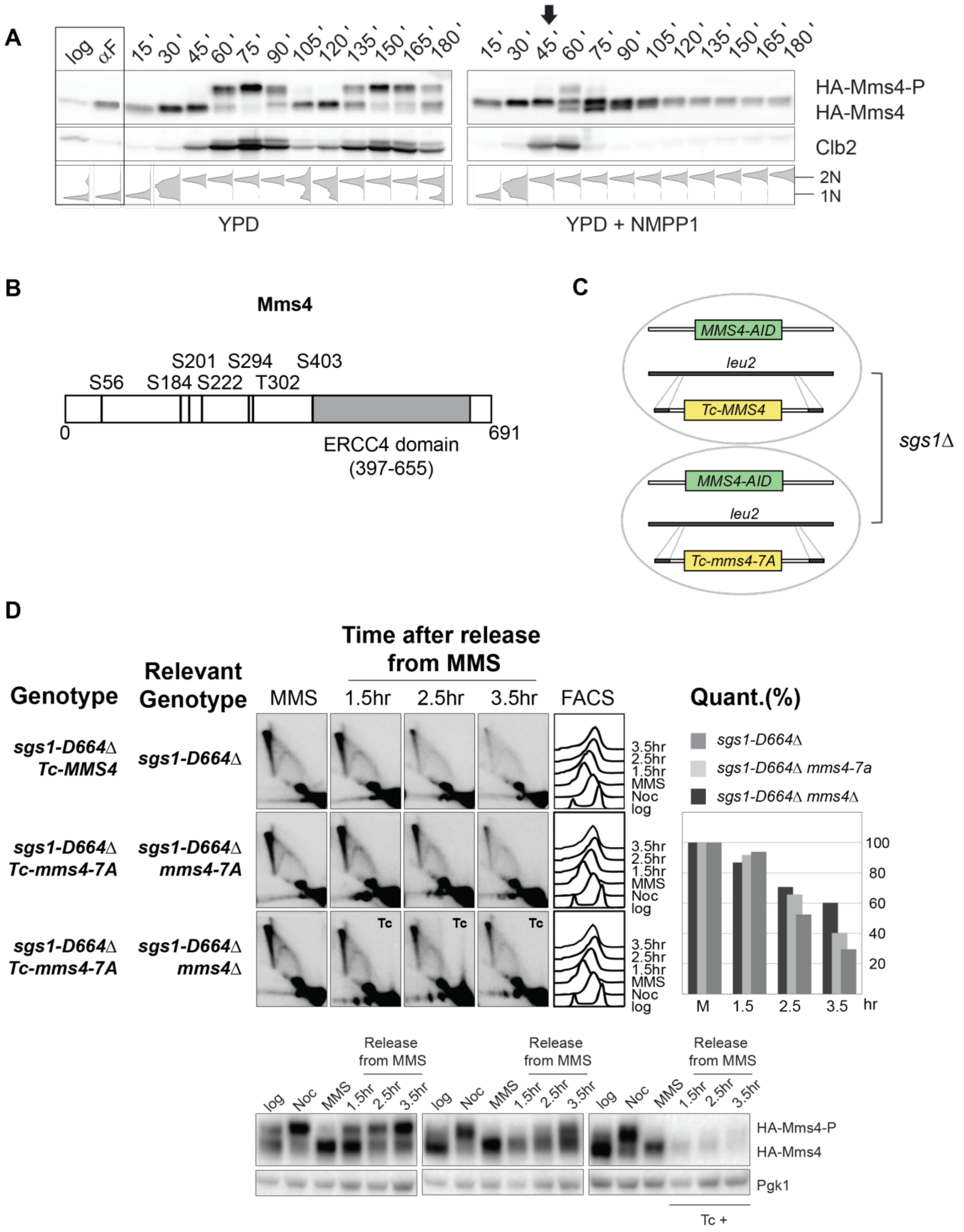

Figure S3. Recurrent G2/M-induced Mms4 phosphorylation is required for the late resolution of DDT intermediates. (**A**) Mms4 is phosphorylated in a Cdk1-dependent manner in the G2/M of the cell cycle. *HA-MMS4 cdc28-as1* (HY2207) cells were synchronized in G1, released into YPD media and divided in two identical sets. The cultures were incubated in YPD media for 45 min in order to allow replication initiation through origin firing and then one set was treated with NMPP1, while the other one was left untreated. The arrow indicates the time of NMPP1 addition.

Samples for FACS and protein analysis were taken at the indicated time points. Mms4 (tagged with 3HA) phosphorylation and Clb2 expression levels were analyzed by Western blot. (**B**) Map of the putative CDK consensus sites present in Mms4. S and T represent the serine and threonine residues mutated to alanine in *mms4-7A*. (**C**) Experimental strategy to establish *sgs1 MMS4-AID Tc-MMS4* and *sgs1 MMS4-AID Tc-mms4-7A* strains related to Figure 2B. *Tc-MMS4* and *Tc-mms4-7A* alleles were integrated at the *LEU2* locus in wild-type cells and the resulting strains were crossed with *sgs1D MMS4-AID* strains. For further details refer to the Extended Experimental Procedures section. (**D**) Mms4 phosphorylation is required for the late resolution. G2 synchronized *sgs1-D664*Δ *Tc-MMS4* (HY3301) and *sgs1D664*Δ *Tc-mms4-7A* (HY2368) cells were released in media containing MMS 0.033% for 90 min and then allowed to recover in YPD media. *sgs1 D664*Δ *Tc-mms4-7A* cultures were divided into two identical sets. One set was treated with tetracycline (Tc) to induce Mms4-7A depletion, whereas the other set remained untreated to reveal the effect of the *mms4-7A* mutation on DDT intermediate processing. Samples were taken at the indicated time points for FACS and 2D gel analysis. For each strain, the value for X-molecule signal accumulated during MMS treatment was considered as 100%.

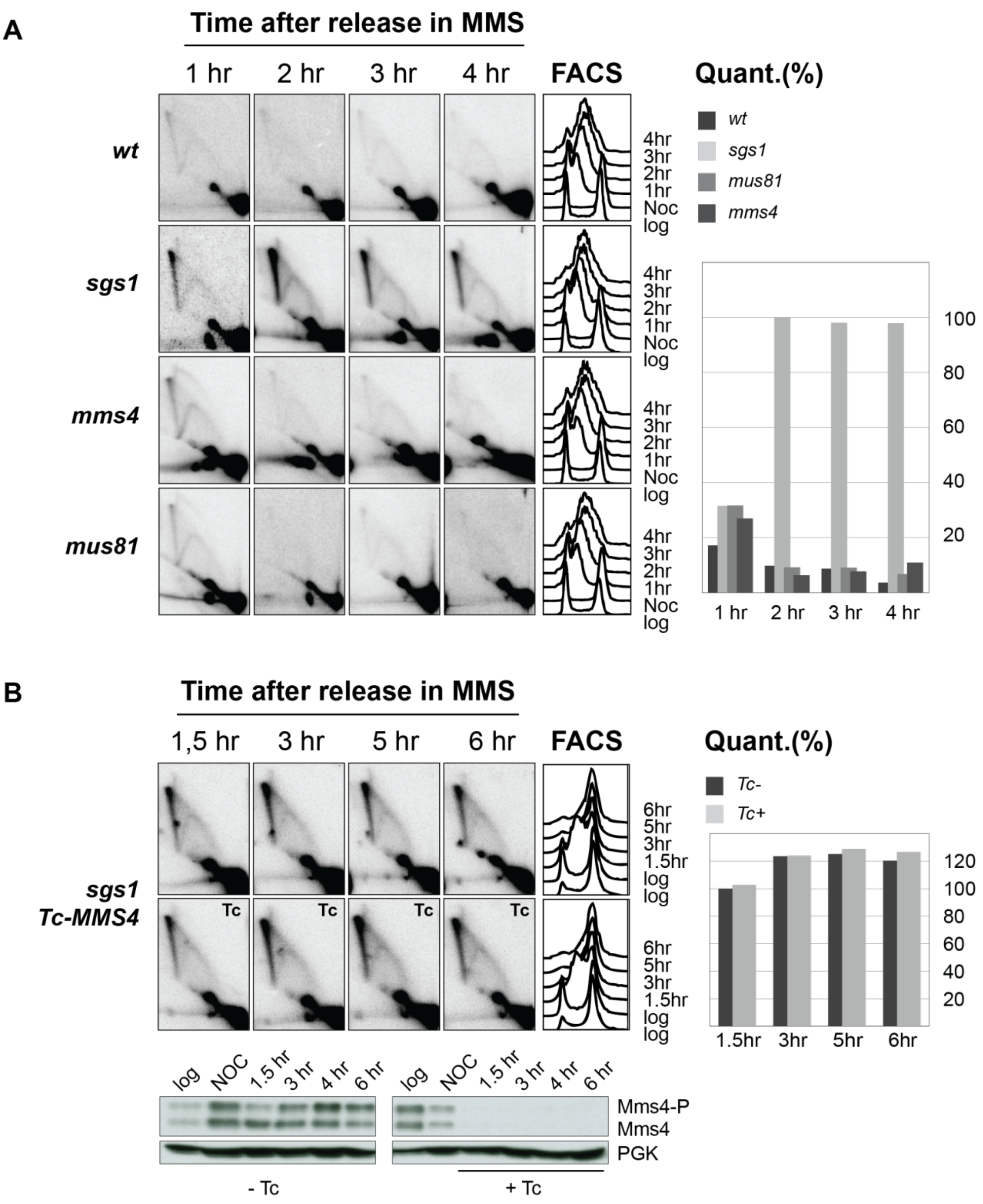

Figure S4. Mus81-Mms4 is not required for template switch intermediate resolution in S-phase. (**A**) Unlike Sgs1, Mus81-Mms4 does not counteract recombination intermediate accumulation during replication of damaged templates. wt (FY0113), *sgs1* (HY0764), *mms4* (HY1958), and *mus81* (HY1475) cells were synchronized in G2, then released in the presence of MMS 0.033%. The highest value for X-molecule signal obtained during quantification was considered as 100%. (**B**) Depletion of Mms4 in *sgs1* mutant cells does not impact on X-molecule accumulation during replication. Logarithmically growing *sgs1 Tc-MMS4* (HY2915) cells were divided

into two sets and treated with 0.033% MMS. Tetracycline (Tc) was added to one set of strains and Mms4 depletion was followed via immunoblotting. For both conditions (with or without tetracycline), the value for X-molecule signal obtained during quantification after 1.5 hour of MMS treatment was considered as 100%.

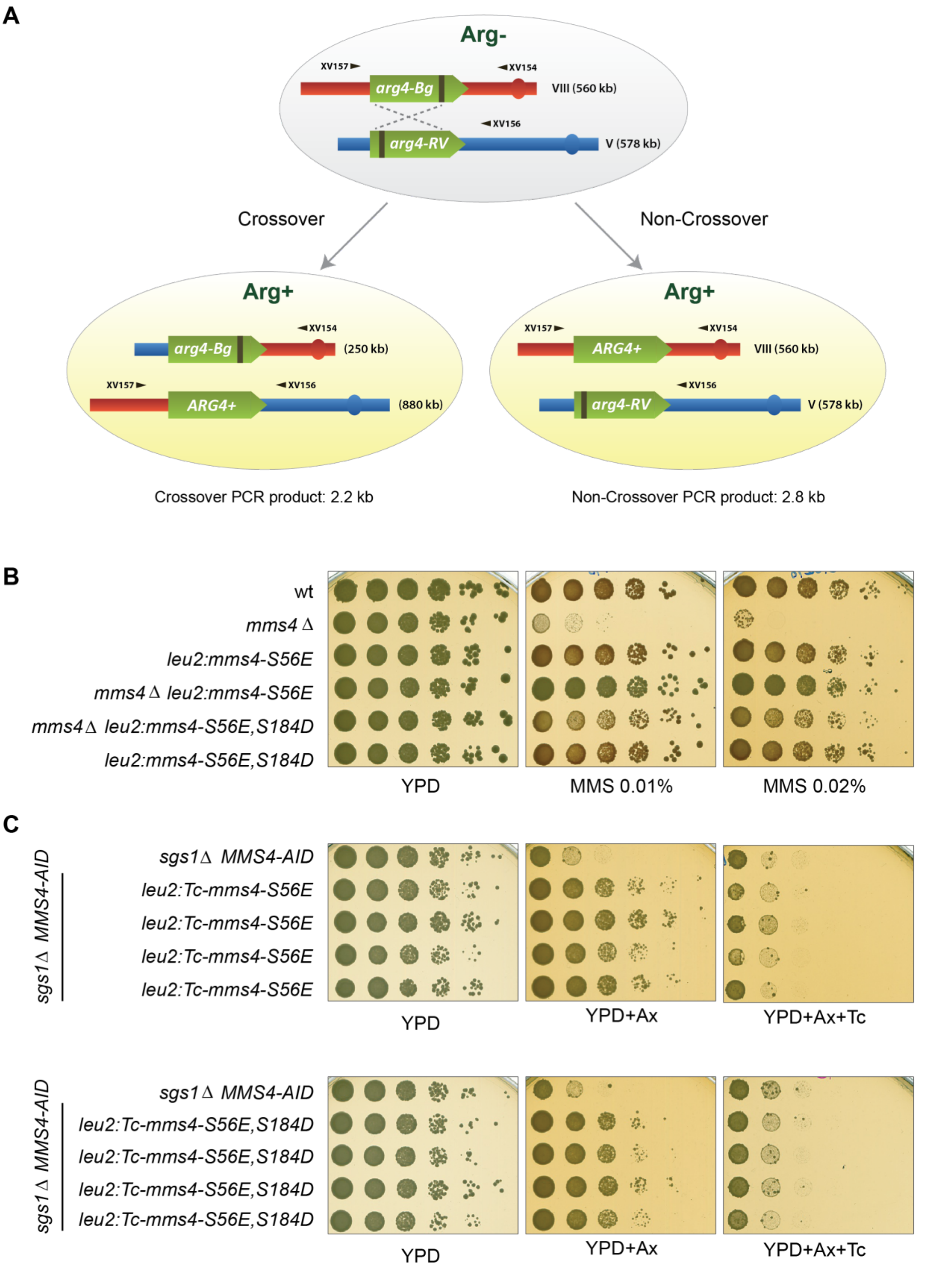

Figure S5. Schematic overview of the crossover assay and genetic characterization of Mms4 phosphorylation mimicking variants. (**A**) Schematics of the crossover assay employed in Figures 4 and 6 as described in (Robert et al, 2006). Crossover frequencies associated to gene conversion events were determined in haploid cells carrying two *arg4* alleles mutated at different sites and located on different chromosomes. One allele, *arg4ΔBglII*, is at its endogenous location on chromosome

VIII, and the other, *arg4ΔEcoRV*, flanked by two alleles of *URA3* was introduced on chromosome V by a pop-in event using the pNM20 plasmid (Robert et al, 2006). Both heteroalleles are in the same orientation with respect to the centromere. Cells that have undergone gene conversion were selected for their ability to grow on synthetic medium lacking arginine. Crossover recombinants were distinguished from non-crossover recombinants by PCR as described in the Extended Experimental Procedures. (**B**) Genetic Characterization of Mms4 Phosphorylation Mimicking Variants. wt (FY1000), *mms4D* (HY1728), *Tc-mms4-S56E* (HY3019), *mms4D Tc-mms4-S56E* (HY3092), *mms4D Tc-mms4-S56E,T184D* (HY3048), *Tc-mms4-S56E,T184D* (HY3021) strains were grown to log phase and analyzed for MMS sensitivity by spot assay. (C) Spot assay measuring the viability of *sgs1 MMS4-AID* (HY3189), *sgs1 MMS4-AID Tc-mms4-S56E* (HY3075, HY3216, HY3076, HY3217) and *Tc-mms4-S56E, T184D* (HY3078, HY3079, HY3181, HY3182) strains in the presence or absence of Auxin (Ax) that induces Mms4-Aid depletion, or in the presence of both Auxin (Ax) and Tetracycline (Tc), which induce depletion of both Mms4-AID and Tc-Mms4-S56E (Tc-Mms4-S56E, S184D) proteins.

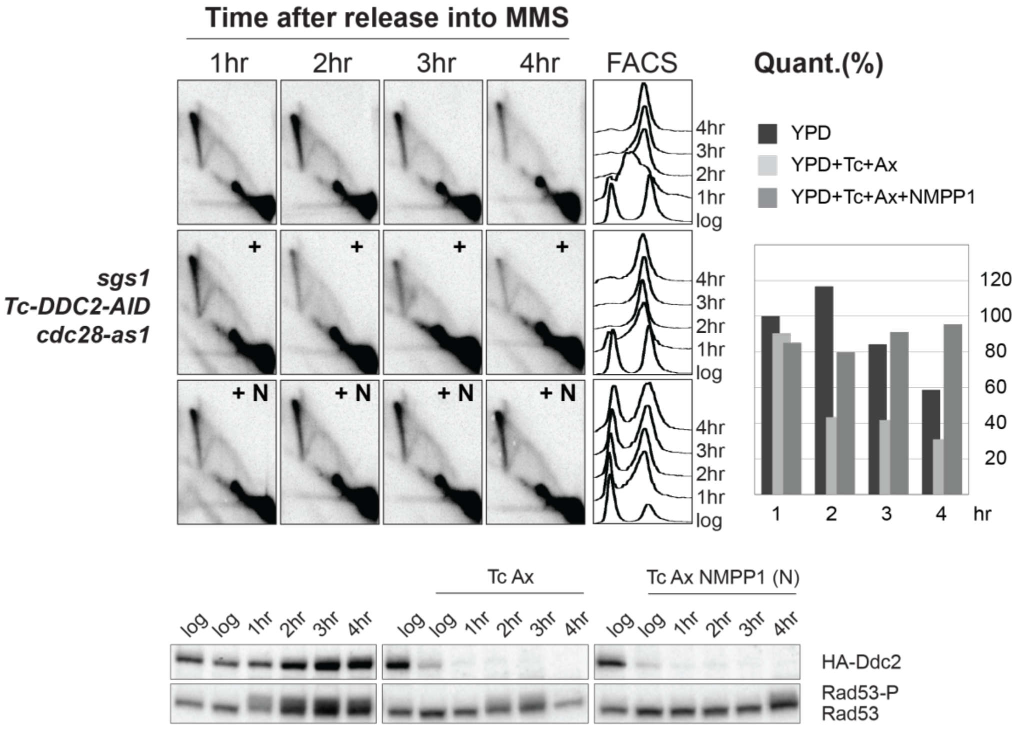

Figure S6. Mec1-Ddc2 counteracts Cdk1-mediated processing of damage-bypass intermediates during replication. Logarithmically growing *sgs1 Tc-DDC2-AID cdc28-as1* (HY2730) cells were released into MMS as before and split into three parts: the first set remained untreated, the second was treated with auxin and tetracycline (+), and the third set was treated with auxin, tetracycline and NMPP1 (+N). At the indicated time-points samples were taken for 2D gel and FACS analysis. The value obtained for the X-molecules accumulating 1h following the release from MMS, in the untreated series, was considered as 100%. Ddc2 depletion (tagged with 3HA) and checkpoint deactivation were analyzed by Western blot using anti-HA and anti-Rad53 (EL7) antibodies respectively.

Table S1. List of strains used in this study. The strains are listed in alphabetical/number ascending order.

FY0113	MATα *his3-Δ200 leu2-3, 112 lys-801 trp1-1 (am) ura3-52*	DF5 wt	Lab strain collection
FY1000	*MAT*a, *ade2-1, trp1-1, leu2-3,112, his3-11-15, ura3, can1-100*	W303 *wt*	Lab strain collection
FY1081	MATa *ade2-1 ura3 trp1-1 leu2-3, 112 his3-11, 15 can1-100 GAL- PSI+ RAD5 esc2Δ::NATMX4*	W303 *esc2Δ*	Lab strain collection
FY1083	MATα *ADE2 trp1 leu2 LYS2 RAD5 sgs1D664Δ*	W303 *sgs1D664Δ*	(Bernstein et al, 2009)
FY1421	Mata *ade2-1 ura3 trp1-1 leu2-3 leu2-112 his3-11 his3-15 can1-100 GAL-PSI+ POL1::POL1-His3FLAG::KANMX4*	W303 wt	Lab strain collection
FY1422	Mata *ade2-1 ura3 trp1-1 leu2-3 leu2-112 his3-11 his3-15 can1-100 GAL-PSI+ POL1::POL1 His3FLAG::KANMX4 CDC28::cdc28-as1::URA3*	W303 *cdc28-as1*	Lab strain collection
FY1485	*MATα arg4ΔBglII CAN1 URA3::arg4ΔEcoRV::ura3-1 RAD5+*	W303 crossover strain *wt*	Lab strain collection
FY1518	MATa *arg4ΔBglII CAN1 URA3::arg4ΔEcoRV::ura3-1 RAD5+ mrc1-AQ-MYC13::HIS3*	W303 crossover strain *mrc1-AQ*	Lab strain collection
FY1519	*MATα arg4ΔBglII CAN1 URA3::arg4ΔEcoRV::ura3-1 RAD5+ rad53Δ::HIS3 sml1-1*	W303 crossover strain *rad53Δ sml1*	Lab strain collection
FY1521	*MATα arg4ΔBglII CAN1 URA3::arg4ΔEcoRV::ura3-1 RAD5+ srs2Δ::LEU2*	W303 crossover strain *srs2Δ*	Lab strain collection
HY0501	MATα *his3-Δ200 leu2-3, 112 lys2-801 trp1-1 (am) ura3-52 sgs1::AUR1-C*	DF5 *sgs1*	Lab strain collection
HY0666	MATα *his3-Δ200 leu2-3, 112 lys-801 trp1-1 (am) ura3-52 sgs1::AUR1-C CDC28::cdc28-as1::URA3*	DF5 *sgs1 cdc28-as1*	This study
HY0764	MATa *his3-Δ200 leu2-3, 112 lys2-801 trp1-1 (am) ura3-52 sgs1Δ::NATMX4*	DF5 *sgs1Δ*	Lab strain collection
HY0992	MATa *ade2-1 trp1-1 leu2-3,112 his3-11-15 ura3 can1-100 rad51Δ::LEU2 sgs1Δ::NATMX4*	W303 *sgs1Δ rad51Δ*	Lab strain collection
HY1475	MATα *his3-Δ200 leu2-3, 112 lys-801 trp1-1 (am) ura3-52 mus81Δ::KANMX4*	DF5 *mus81Δ*	This study
HY1728	MATa *ade2-1, trp1-1, leu2-3,112, his3-11-15, ura3, can1-100 RAD5 mms4Δ::HPHMX4*	W303 *mms4Δ*	This study
HY1947	MATa *ade2-1 ura3 trp1-1 leu2-3, 112 his3-11, 15 can1-100 GAL-PSI+ RAD5 sgs1Δ::HIS3 mms4::pADH1-tc3-3xHA-*	W303 *sgs1Δ Tc-MMS4*	This study

	MMS4:: KANMX4		
HY1958	MATα *his3-Δ200 leu2-3, 112 lys-801 trp1-1 (am) ura3-52 mms4Δ::HPHMX4*	DF5 *mms4Δ*	This study
HY2039	MATa *his3-Δ200 leu2-3, 112 lys2-801 trp1-1 (am) ura3-52 sgs1Δ::HPHMX4 clb2Δ::NATMX4*	DF5 *sgs1Δ clb2Δ*	This study
HY2055	MATa *ade2-1 ura3 trp1-1 leu2-3, 112 his3-11, 15 can1-100 GAL-PSI+ RAD5 esc2Δ::NATMX4 yen1Δ::HIS3*	W303 *esc2Δ yen1Δ*	This study
HY2207	MATa *ade2-1 ura3 trp1-1 leu2-3 leu2-112 his3-11 his3-15 can1-100 GAL PSI+ RAD5 pADH1-tc3-3xHA-MMS4::KANMX4 CDC28::cdc28-as1::URA3*	W303 *Tc-MMS4 cdc28-as1*	This study
HY2280	*MATα arg4ΔBglII CAN1 URA3::arg4ΔEcoRV::ura3-1 RAD5+ rad53Δ::HIS3 sml1-1 mms4Δ::HPHMX4*	W303 crossover strain *rad53Δ sml1 mms4Δ*	This study
HY2367	MATalfa *ade2-1 ura3 trp1-1 leu2-3 leu2-112 his3-11 his3-15 can1-100 GAL-PSI+ RAD5 sgs1D664Δ mms4::pADH-Tc3-3xHA-mms4-7A::KANMX4*	W303 *sgs1D664Δ Tc-mms4-7A*	This study
HY2368	MATα *ADE2 trp1 leu2 LYS2 RAD5 sgs1-D664Δ mms4::pADH-Tc3-3xHA-mms4-7A::KANMX4*	W303 *sgs1D664Δ Tc-mms4 -7A*	This study
HY2395	MATa *ura3-1::ADH1-OsTIR1-9Myc(URA3) ade2-1 his3-11,15 leu2-3,112 trp1-1 can1-100 sgs1Δ::NATMX4 ddc2:: pADH1-Tc3-3xHA(KANMX)-DDC2-AID(HPHMX4)*	W303 *sgs1Δ Tc-DDC2-AID*	This study
HY2443	MATa *ura3-1::ADH1-OsTIR1-9Myc(URA3) ade2-1 his3-11,15 leu2-3,112 trp1-1 can1-100 sgs1Δ::NATMX4 ddc2:: pADH1-tc3-3xHA(kanMX)-DDC2-AID(HPHMX4) tel1Δ::HIS3*	W303 *sgs1Δ Tc-Ddc2-AID tel1Δ*	This study
HY2640	MATa ade2-1 ura3-1 his3-11,15 trp1-1 leu2-3,112 can1-100 ura3-1::ADH1-OsTIR1-9Myc (URA3) sgs1*Δ*::HIS3 mms4::MMS4-AID(KANMX) leu2::pADH-tc3-3xHA-MMS4::KANMX4	W303 sgs1*Δ* MMS4-AID leu2::Tc-MMS4	This study
HY2642	MATa ade2-1 ura3-1 his3-11,15 trp1-1 leu2-3,112 can1-100 ura3-1::ADH1-OsTIR1-9Myc (URA3) sgs1*Δ*::HIS3 mms4::MMS4-AID(KANMX) leu2::pADH-tc3-3xHA-mms4-7A::KANMX4	W303 sgs1*Δ* MMS4-AID leu2::Tc-mms4-7A	This study
HY2693	*MATa arg4ΔBglII CAN1 URA3::arg4ΔEcoRV::ura3-1 RAD5+ mms4Δ::HPHMX4 lleu2::pADH-Tc3-*	W303 crossover strain *Tc-MMS4*	This study

	3xHA-MMS4::KanMX4		
HY2730	MATa *ura3-1::ADH1-OsTIR1-9Myc(URA3) ade2-1 his3-11,15 leu2-3,112 trp1-1 can1-100 ddc2:: pADH1-Tc3-3xHA(KANMX)-DDC2-AID(HPHMX4) sgs1Δ::NATMX CDC28::cdc28-as1::URA3*	W303 *sgs1Δ Tc-DDC2-AID cdc28-as1*	This study
HY2861	MATa *ura3-1::ADH1-OsTIR1-9Myc(URA3) ade2-1 his3-11,15 leu2-3,112 trp1-1 can1-100 ddc2:: pADH1-Tc3-3xHA(KANMX)-DDC2-AID(HPHMX4) sgs1Δ::HIS3 pADH1-Tc3-3xHA-MMS4::KanMX4*	W303 *sgs1Δ Tc-DDC2-AID Tc-MMS4*	This study
HY2915	MATa *ADE2+ CAN1+ ura3-1 his3-11 leu2-3, 112 trp1-1 RAD5+ DUN1::DUN1-3HA-TRP1 GAL-rad53D339A::LEU2 sgs1Δ::HPHMX4 pADH1-tc3-3xHA-MMS4::KANMX4*	W303 *sgs1Δ GAL-rad53-D339A Tc-MMS4*	This study
HY3019	MATa *ade2-1 trp1-1 leu2-3,112 his3-11,15 ura3 can1-100 leu2::pADH-Tc3-3HA-mms4-S56E::KanMX4*	W303 *leu2::Tc-mms4-S56E*	This study
HY3021	*MATa ade2-1 trp1-1 leu2-3,112 his3-11,15 ura3 can1-100 leu2::pADH-Tc3-3HA-mms4-S56E, 184D::KanMX4*	W303 *leu2::Tc-mms4-S56E,T184D*	This study
HY3048	*MATa ade2-1 trp1-1 leu2-3112 his3-1115 ura3 can1-100 GAL PSI1+ RAD5+ leu2::pADH-Tc3-3HA-mms4-S56E,T184D::kanMX4 mms4Δ::HPHMX4*	W303 *mms4Δ leu2::Tc-mms4-S56E,T184D*	This study
HY3058	MATa *ade2-1 trp1-1 leu2-3112 his3-1115 ura3 can1-100 GAL PSI1+ RAD5+ sgs1Δ::HIS3 cdc5-as1*	W303 *sgs1Δ cdc5-as1*	This study
HY3075	*MATa ade2-1 trp1-1 leu2-3112 his3-1115 ura3-1::ADH1-OsTIR1-9Myc::URA3 sgs1Δ::NATMX4 mms4::MMS4-AID::HPHMX4 leu2::pADH1-Tc3-3xHA-mms4-S56E::KanMX4*	W303 *sgs1Δ MMS4-AID leu2::Tc-mms4-S56E*	This study
HY3076	MATa *ade2-1 trp1-1 leu2-3112 his3-1115 ura3-1::ADH1-OsTIR1-9Myc::URA3 sgs1Δ::NATMX4 mms4::MMS4-AID::HPHMX4 leu2::pADH1-Tc3-3xHA-mms4-S56E::KanMX4*	W303 *sgs1Δ MMS4-AID leu2::Tc-mms4-S56E*	This study
HY3078	MATa *ade2-1 trp1-1 leu2-3112 his3-1115 ura3-1::ADH1-OsTIR1-9Myc::URA3 sgs1Δ::NATMX4 mms4::MMS4-AID::HPHMX4 leu2::pADH1-tc3-3xHA-mms4-S56E,T184D::KanMX4*	W303 *sgs1Δ MMS4-AID leu2::Tc-mms4-S56E,184D*	This study
HY3079	MATa *ade2-1 trp1-1 leu2-3112 his3-1115 ura3-1::ADH1-OsTIR1-9Myc::URA3 sgs1Δ::NATMX4 mms4::MMS4-AID::HPHMX4*	W303 *sgs1Δ MMS4-AID leu2::Tc-mms4-S56E,184D*	This study

	leu2::pADH1-tc3-3xHA-mms4-S56E,T184D::KanMX4		
HY3092	*MATa, ade2-1, trp1-1, leu2-3112, his3-1115, ura3, can1-100 GAL PSI1+ RAD5+ mms4Δ::HPHMX4 leu2::pADH-Tc3-3HA-mms4-S56E::kanMX4*	W303 *mms4Δ leu2::Tc-mms4-S56E*	This study
HY3150	*MATα ade2-1 trp1-1 leu2-3112 his3-1115 ura3 can1-100 GAL PSI1+ RAD5+ pADH1-tc3-3xHA-MMS4::KanMX4 sgs1Δ::HIS3 cdc5-as1*	W303 *sgs1Δ cdc5-as1 Tc-MMS4*	This study
HY3181	MATa *ade2-1 trp1-1 leu2-3112 his3-1115 ura3-1::ADH1-OsTIR1-9Myc::URA3 sgs1Δ::NATMX4 mms4::MMS4-AID::HPHMX4 leu2::pADH1-tc3-3xHA-mms4-S56E,S184D::KanMX4*	W303 *sgs1Δ MMS4-AID leu2::Tc-mms4-S56E,184D*	This study
HY3182	MATa *ade2-1 trp1-1 leu2-3112 his3-1115 ura3-1::ADH1-OsTIR1-9Myc::URA3 sgs1Δ::NATMX4 mms4::MMS4-AID::HPHMX4 leu2::pADH1-tc3-3xHA-mms4-S56E,S184D::KanMX4*	W303 *sgs1Δ MMS4-AID leu2::Tc-mms4-S56E,184D*	This study
HY3189	*MATa ade2-1 trp1-1 leu2-3112 his3-1115 ura3-1::ADH1-OsTIR1-9Myc::URA3 sgs1Δ::NATMX4 mms4::MMS4-AID::HPHMX4*	W303 *sgs1Δ MMS4-AID*	This study
HY3216	MATa *ade2-1 trp1-1 leu2-3112 his3-1115 ura3-1::ADH1-OsTIR1-9Myc::URA3 sgs1Δ::NATMX4 mms4::MMS4-AID::HPHMX4 leu2::pADH1-Tc3-3xHA-mms4-S56E::KanMX4*	W303 *sgs1Δ MMS4-AID leu2::Tc-mms4-S56E*	This study
HY3217	MATa *ade2-1 trp1-1 leu2-3112 his3-1115 ura3-1::ADH1-OsTIR1-9Myc::URA3 sgs1Δ::NATMX4 mms4::MMS4-AID::HPHMX4 leu2::pADH1-Tc3-3xHA-mms4-S56E::KanMX4*	W303 *sgs1Δ MMS4-AID leu2::Tc-mms4-S56E*	This study
HY3244	*MATα ADE2 trp1 leu2 LYS2 RAD5 sgs1-D664Δ slx1::HPHMX4*	W303 *sgs1D664Δ* slx1*Δ*	This study
HY3247	*MATα arg4ΔBglII CAN1 URA3::arg4ΔEcoRV::ura3-1 RAD5+ srs2Δ::LEU2 mms4Δ::HPHMX4 leu2::pADH-Tc3-3xHA-MMS4::KanMX4*	W303 crossover strain *srs2Δ mms4Δ Tc-MMS4*	This study
HY3250	*MATa arg4ΔBglII CAN1 URA3::arg4ΔEcoRV::ura3-1 RAD5+ srs2Δ::LEU2 mms4Δ::HPHMX4 leu2::pADH-Tc3-3xHA-mms4-7A::KanMX4*	W303 crossover strain *srs2Δ mms4Δ Tc-mms4-7A*	This study
HY3256	*MATα arg4ΔBglII CAN1 URA3::arg4ΔEcoRV::ura3-1 RAD5+ srs2Δ::LEU2 mms4Δ::HPHMX4*	W303 crossover strain *srs2Δ mms4Δ*	This study

HY3260	*MATα arg4ΔBglII CAN1 URA3::arg4ΔEcoRV::ura3-1 RAD5+ mms4Δ::HPHMX4 leu2::pADH-Tc3-3xHA-mms4-56E::KanMX4*	W303 crossover strain *Tc-mms4-S56E*	This study
HY3264	*MATα arg4ΔBglII CAN1 URA3::arg4ΔEcoRV::ura3-1 RAD5+ mms4Δ::HPHMX4 leu2::pADH-Tc3-3xHA-mms4-56E,T184D::KanMX4*	W303 crossover strain *Tc-mms4-S56E,T184D*	This study
HY3301	*MATα ade2-1 ura3 trp1-1 leu2-3, 112 his3-11, 15 can1-100 GAL-PSI+ RAD5 sgs1D664Δ mms4::pADH1-Tc3-3xHA-MMS4::KanMX4*	W303 *sgs1D664Δ Tc-MMS4*	This study
HY3316	MATa *ade2-1 trp1-1 leu2-3112 his3-1115 ura3 can1-100 GAL PSI1+ RAD5+ sgs1Δ::HIS3 cdc5-as1 leu2::pADH1-tc3-3xHA-mms4-S56E::KanMX4*	W303 *sgs1Δ cdc5-as1 Tc-mms4-S56E::Leu2*	This study
HY3318	MATa *ade2-1 trp1-1 leu2-3112 his3-1115 ura3 can1-100 GAL PSI1+ RAD5+ sgs1Δ::HIS3 cdc5-as1 leu2::pADH1-tc3-3xHA-mms4-* S56E,184D::KanMX4	W303 *sgs1Δ cdc5-as1 Tc-mms4-S56E,T184D::Leu2*	This study
HY3332	MATa *ura3-1::ADH1-OsTIR1-9Myc(URA3) ade2-1 his3-11,15 leu2-3,112 trp1-1 can1-100 ddc2:: pADH1-Tc3-3xHA(KANMX)-DDC2-AID(HPHMX4) sgs1Δ*::*NATMX MMS4-PK9::TRP*	W303 *sgs1Δ Tc-DDC2-AID MMS4-PK*	This study
HY3451	MATa *ura3-1::ADH1-OsTIR1-9Myc(URA3) ade2-1 his3-11,15 leu2-3,112 trp1-1 can1-100 ddc2:: pADH1-Tc3-3xHA(KANMX)-DDC2-AID(HPHMX4) sgs1Δ::HIS3 cdc5-as1*	W303 *sgs1Δ Tc-DDC2-AID cdc5-as1*	This study
HY3741	MATa *arg4ΔBglII CAN1 URA3::arg4ΔEcoRV::ura3-1 RAD5+ mrc1-AQ-MYC13::HIS3* mms4Δ::HPHMX4	W303 crossover strain *mrc1-AQ mms4Δ*	This study

Extended Experimental Procedures

Yeast techniques

S. cerevisiae strains were prepared by genetic crosses and standard disruption techniques (Goldstein & McCusker, 1999; Wach et al, 1994). *Tc-MEC1, Tc-DDC2, TC-RAD53* and *Tc-MMS4* conditional alleles were established by integrating cassettes amplified from pADH1-tc3-6xHA (P30599) and pADH1-tc3-3xHA (P30598) (EUROSCARF) plasmids respectively as described in (Kotter et al, 2009). *MMS4*, *DDC2* and *RAD53* genes were tagged with AID in a wild type strain as previously described (Nishimura et al, 2009). Oligo information is available upon request. To construct the *cdc28-as1* allele, plasmid pVF6 (CB1191), generously provided by M. Foiani, was digested with *Cla*I and integrated at the *CDC28* locus. The correct integrations were checked by PCR and by spot assays when the resulted alleles were expected to lead to damage sensitivity or growth defects phenotypes.

To generate tagged and phosphorylation-defective alleles of *MMS4*, the *Tc-3HA-MMS4* allele from a *Tc-MMS4* containing strain (HY1947) was amplified by PCR and inserted into the *Eco*RI site of YIplac211 (Gietz & Sugino, 1988) to obtain pTc-3HA-MMS4. pTc-3HA-mms4-7A (S56A, S184A, S201A, S222A, S294A, T302A, S403A) was generated by multisite-directed mutagenesis (Stratagene) of pTc-3HA-MMS4 using oligos designed by Quick-change software (Agilent Technologies). The *Eco*RI fragment carrying *Tc-3HA-MMS4* or *Tc-3HA-mms4-7A* sequences were integrated into the *MMS4* locus of *mms4*Δ *sgs1D664*Δ strains to generate *sgs1D664*Δ *Tc-MMS4* and *sgs1D664*Δ *Tc-mms4-7A* strains respectively. To obtain *sgs1Δ Tc-MMS4 MMS4-AID* and *sgs1Δ Tc-mms4-7A MMS4-AID* strains, *MMS4* gene was first tagged with AID in a wild type strain as described (Nishimura et al, 2009). *Tc-MMS4* and *Tc-mms4-7A* fragments having sequence homology at the flanking regions to the *LEU2* gene were amplified and integrated into the *LEU2* locus of *MMS4-AID* strain.

Drug treatments

MMS was added directly to cultures to a final concentration of 0.033% unless otherwise indicated. NMPP1 was dissolved in DMSO to a final concentration of 20 mM and added directly to cultures to a final of 5 μM final. CMK-Cl was dissolved in DMSO to a final concentration of 10 mM and added directly to cultures to a final of 5 μM final. In the control samples an identical volume of DMSO solution was added

instead. Auxin and tetracycline were used at 0.5 mM and 0.6 mM concentrations, respectively.

Synchronization by α-factor or nocodazole

Unless otherwise indicated, strains were grown at 25ºC in YP-media containing glucose (2%), YPD, as carbon source. Cells were synchronized either in metaphase by adding nocodazole to a final concentration of 10 μg/ml together with DMSO (SIGMA) to a final of 1% v/v, for about 2.5 hr, or in G1 with α-factor (SIGMA or PRIMM) to a final concentration of 3-5 μg/ml. The release from the synchronization was performed by washing cells twice in YP media, followed by suspension of cells in YPD media containing MMS at a final concentration of 0.033% v/v. For the recovery experiments, following the MMS pulse, cells were released into the cell cycle by washing twice the cells with YP pre-warmed at 30ºC. Following the synchronization step, the experiments were conducted at 30ºC.

FACS analysis

For FACS analysis, approximately $2x10^7$ cells for each time-point were collected, washed in sterile water, and permeabilized in 70% ethanol. Cells were suspended in 10 mM Tris pH 7.5 buffer, and RNA and proteins were removed by RNaseA (0.4 mg/ml final concentration) and proteinase K (1 mg/ml) treatment (SIGMA-ALDRICH). Subsequently, cells were stained in SYTOX green (1 μM) (Invitrogen) solution or propidium iodide (50 μg/ml) (SIGMA-ALDRICH).

TCA protein extraction

Proteins were analyzed from denatured yeast crude extracts as previously described (Liberi et al, 2000). Briefly, about 10^8 cells were collected, washed with 20% TCA and re-suspended in 100 μl TCA 20%. An equal volume of glass beads was added and the suspension was vortexed for 5 minutes, following which the extracts were transferred to a fresh tube. The glass beads were washed twice with 100 μl of TCA 5% and the washings were combined with the crude extract. The pellet of proteins was obtained by centrifugation at 3000 rpm and suspended in about 100 μl of Laemmli loading buffer and neutralized with 1M Tris. The samples were boiled for 3 min, centrifuged at 3000 rpm for 10 min, and the supernatant kept for analysis by Western blotting.

References

Bernstein KA, Shor E, Sunjevaric I, Fumasoni M, Burgess RC, Foiani M, Branzei D, Rothstein R (2009) Sgs1 function in the repair of DNA replication intermediates is separable from its role in homologous recombinational repair. *The EMBO journal* **28:** 915-925

Gietz RD, Sugino A (1988) New yeast-Escherichia coli shuttle vectors constructed with in vitro mutagenized yeast genes lacking six-base pair restriction sites. *Gene* **74:** 527-534

Goldstein AL, McCusker JH (1999) Three new dominant drug resistance cassettes for gene disruption in Saccharomyces cerevisiae. *Yeast* **15:** 1541-1553

Kotter P, Weigand JE, Meyer B, Entian KD, Suess B (2009) A fast and efficient translational control system for conditional expression of yeast genes. *Nucleic Acids Res* **37:** e120

Liberi G, Chiolo I, Pellicioli A, Lopes M, Plevani P, Muzi-Falconi M, Foiani M (2000) Srs2 DNA helicase is involved in checkpoint response and its regulation requires a functional Mec1-dependent pathway and Cdk1 activity. *Embo J* **19:** 5027-5038

Nishimura K, Fukagawa T, Takisawa H, Kakimoto T, Kanemaki M (2009) An auxin-based degron system for the rapid depletion of proteins in nonplant cells. *Nat Methods* **6:** 917-922

Robert T, Dervins D, Fabre F, Gangloff S (2006) Mrc1 and Srs2 are major actors in the regulation of spontaneous crossover. *Embo J* **25:** 2837-2846

Wach A, Brachat A, Pohlmann R, Philippsen P (1994) New heterologous modules for classical or PCR-based gene disruptions in Saccharomyces cerevisiae. *Yeast* **10:** 1793-1808
